# Unobtrusive Sensing and Wearable Devices for Health Informatics

**DOI:** 10.1109/TBME.2014.2309951

**Published:** 2014-03-05

**Authors:** Ya-Li Zheng, Xiao-Rong Ding, Carmen Chung Yan Poon, Benny Ping Lai Lo, Heye Zhang, Xiao-Lin Zhou, Guang-Zhong Yang, Ni Zhao, Yuan-Ting * Zhang

**Affiliations:** 1 Department of Electronic EngineeringJoint Research Centre for Biomedical Engineering, The Chinese University of Hong Kong Hong Kong; 2 Department of SurgeryThe Chinese University of Hong Kong Hong Kong; 3 Department of ComputingImperial College London London SW7 2AZ U.K.; 4 Institute of Biomedical and Health Engineering, Chinese Academy of Sciences (CAS)SIAT Shenzhen China; 5 Key Laboratory for Health Informatics of the Chinese Academy of Sciences (HICAS)SIAT Shenzhen 518055 China; 6 Department of Electronic EngineeringThe Chinese University of Hong Kong Hong Kong

**Keywords:** Body sensor network, flexible and stretchable electronics, health informatics, sensor fusion, unobtrusive sensing, wearable devices

## Abstract

The aging population, prevalence of chronic diseases, and outbreaks of infectious diseases are some of the major challenges of our present-day society. To address these unmet healthcare needs, especially for the early prediction and treatment of major diseases, health informatics, which deals with the acquisition, transmission, processing, storage, retrieval, and use of health information, has emerged as an active area of interdisciplinary research. In particular, acquisition of health-related information by unobtrusive sensing and wearable technologies is considered as a cornerstone in health informatics. Sensors can be weaved or integrated into clothing, accessories, and the living environment, such that health information can be acquired seamlessly and pervasively in daily living. Sensors can even be designed as stick-on electronic tattoos or directly printed onto human skin to enable long-term health monitoring. This paper aims to provide an overview of four emerging unobtrusive and wearable technologies, which are essential to the realization of pervasive health information acquisition, including: 1) unobtrusive sensing methods, 2) smart textile technology, 3) flexible-stretchable-printable electronics, and 4) sensor fusion, and then to identify some future directions of research.

## I. Introduction

Global healthcare systems are struggling with aging population, prevalence of chronic diseases, and the accompanying rising costs [1]. In response to these challenges, researchers have been actively seeking for innovative solutions and new technologies that could improve the quality of patient care meanwhile reduce the cost of care through early detection/intervention and more effective disease/patient management. It is envisaged that the future healthcare system should be preventive, predictive, preemptive, personalized, pervasive, participatory, patient-centered, and precise, i.e., p-health system.

**Fig. 1.**
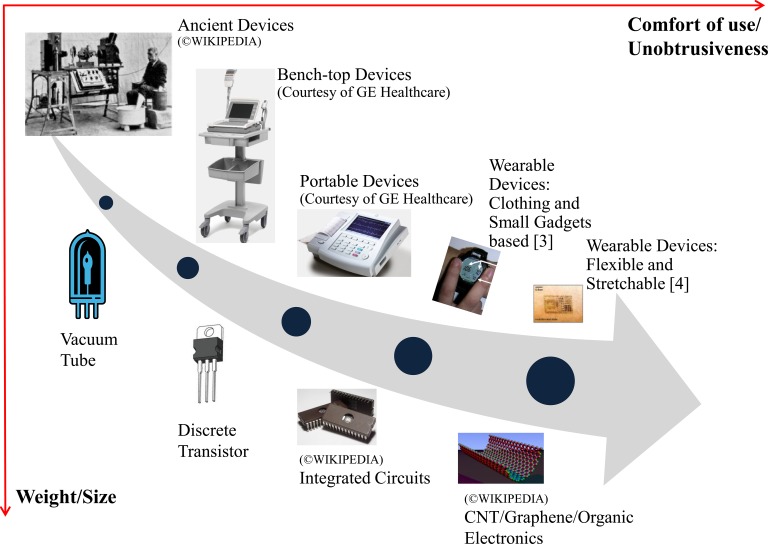
Timelines of medical devices for ECG measurement with the evolution of electronic technology.

Health informatics, which is an emerging interdisciplinary area to advance p-health, mainly deals with the acquisition, transmission, processing, storage, retrieval, and use of different types of health and biomedical information [2]. The two main acquisition technologies of health information are sensing and imaging. This paper focuses only on sensing technologies and reviews the latest developments in unobtrusive sensing and wearable devices for continuous health monitoring. Looking back in history, it is not surprised to notice that innovation in this area is closely coupled with the advancements in electronics. Using electrocardiogram (ECG) device as an example, Fig. 1 illustrates the evolution of sensing technologies, where the core technologies of these devices have evolved from water buckets and bulky vacuum tubes, bench-top, and portable devices with discrete transistors, to the recent clothing and small gadgets based wearable devices with integrated circuits [3]. In the future, it may evolve into flexible and stretchable wearable devices with carbon nanotube (CNT)/graphene/organic electronics [4]. There is a clear trend that the devices are getting smaller, lighter, and less obtrusive and more comfortable to wear.

Although physiological measurement devices have been widely used in clinical settings for many years, some unique features of unobtrusive and wearable devices due to the recent advances in sensing, networking and data fusion have transformed the way that they were used in. First, with their wireless connectivity together with the widely available internet infrastructure, the devices can provide real-time information and facilitate timely remote intervention to acute events such as stroke, epilepsy and heart attack, particularly in rural or otherwise underserved areas where expert treatment may be unavailable. In addition, for healthy population, unobtrusive and wearable monitoring can provide detailed information regarding their health and fitness, e.g., via mobile phone or flexible displays, such that they can closely track their wellbeing, which will not only promote active and healthy lifestyle, but also allow detection of any health risk and facilitate the implementation of preventive measures at an earlier stage.

The objectives of this paper are to provide an overview of unobtrusive sensing and wearable systems with particular focus on emerging technologies, and also to identify the major challenges related to this area of research. The rest of this paper is organised as follows: Section II will discuss unobtrusive sensing methods and systems. Section III will present the recent advances and core technologies of wearable devices and will also highlight the latest developments in smart textile technology and flexible-stretchable electronics. Section IV will discuss data fusion methods for sensing informatics as well as the impacts of big data in healthcare. Section V will conclude the paper with promising directions for future research.

## II. Unobtrusive Sensing Methods and Systems

**Fig. 2.**
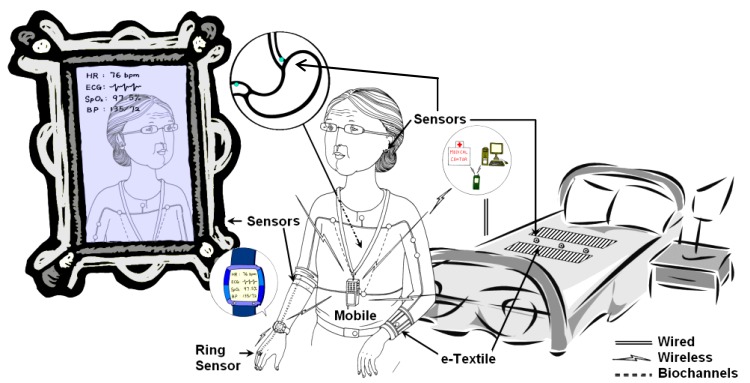
Illustration of unobtrusive physiological measurements in a home environment [5].

**Fig. 3.**
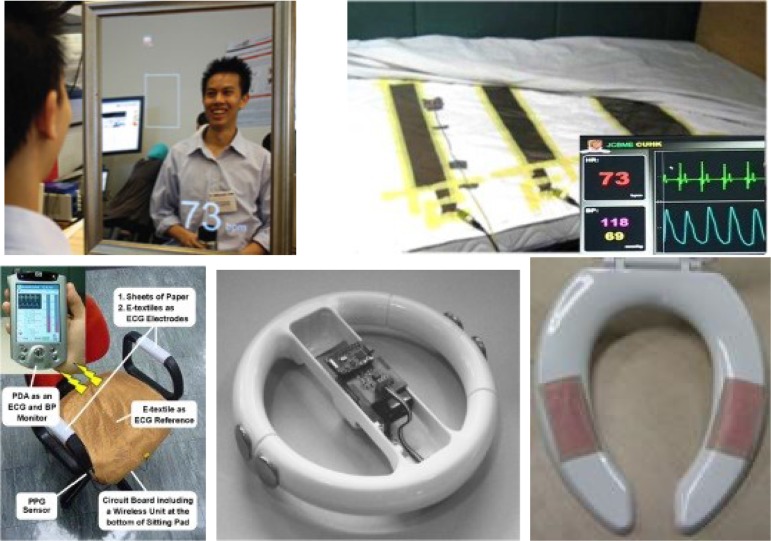
Unobtrusive sensing devices with sensors embedded in daily objects, such as mirror [10], sleeping bed [9], chair [6], steering wheel [11], and toilet seat [13].

The main objective of unobtrusive sensing is to enable continuous monitoring of physical activities and behaviors, as well as physiological and biochemical parameters during the daily life of the subject. The most commonly measured vital signs include: ECG, ballistocardiogram (BCG), heart rate, blood pressure (BP), blood oxygen saturation (SpO_2_), core/surface body temperature, posture, and physical activities. A conceptual model of unobtrusive physiological monitoring in a home setting is shown in Fig. 2 [5]. Unobtrusive sensing can be implemented in two ways: 1) sensors are worn by the subject, e.g., in the form of shoes, eyeglasses, ear-ring, clothing, gloves and watch, or 2) sensors are embedded into the ambient environment or as smart objects interacting with the subjects, e.g., a chair [6], [7], car seat [8], mattress [9], mirror [10], steering wheel [11], mouse [12], toilet seat [13], and bathroom scale [14]. Fig. 3 shows some prototypes of the unobtrusive sensing systems developed by different research groups. Information can be collected by a smartphone and transmitted wirelessly to a remote center for storage and analysis. In the following section, we will discuss some unobtrusive sensing methods for the acquisition of vital signs.

### A. Capacitive Sensing Method

Capacitance-coupled sensing method is commonly used for measuring biopotentials such as ECG, electroencephalogram (EEG) and electromyogram (EMG) [7], [15]. For this method, the skin and the electrode form the two layers of a capacitor. Without direct contact with the body, some issues, such as skin infection and signal deterioration, brought about by adhesive electrodes in long term monitoring can be avoided. Some typical implementations of capacitive ECG sensing are summarized in Table I. In addition, capacitive sensing can also be used for other applications such as respiratory measurement, e.g., using a capacitive textile force sensor weaved into clothing [16], or a capacitive electrical field sensor array placed under a sleeping mattress [17]. The major challenges in designing these noncontact electrodes lie in the high contact impedance due to the indirect contact and the capacitive mismatch caused by motion artifacts [15]. It may cause low signal to noise ratio and thus lead to challenges in the front-end analog circuit design. The input impedance of the amplifier has to be extremely large (>1 TΩ) to reduce the shunt effect formed by the capacitor and the input impedance. Moreover, motion artifacts may become more significant in noncontact sensing. Some methods have recently been proposed to overcome these problems to achieve robust measurement in practical situations. For instance, the gradiometric measurement technique introduced by Pang et al. can considerably reduce motion artifacts [24].

**TABLE I:** Different Implementations of Capacitive ECG Sensors

Systems	Location of ECG electrodes	Measured signals and parameters
Sleeping bed [9]	Bed cushion	ECG, PPG, Heart rate, BP
Wearable ECG system [18]	Cloth and belt	ECG
BP monitoring Chair [6]	Chair pad and arms	ECG, PPG, heart rate, BP
Non-contact chair based system [7]	Chair back	ECG, BCG, heart rate, BP
Aachen Smart Chair [19]	Chair backrest and pad	ECG
Ambulatory ECG monitoring over cloth [20]	Integrated on underwear	ECG
Textile integrated long-term ECG monitor [21]	Integrated into garment	ECG
Non-contact ECG/EEG electrodes [22]	Embedded within fabric and clothing	EEG, ECG
Wireless Wearable ECG sensor [23]	Integrated into a cotton T-shirt	ECG

### B. Photoplethysmographic Sensing Method

Photoplethysmographic (PPG) sensing, which involves a light source to emit light into tissue and a photo-detector to collect light reflected from or transmitted through the tissue, has been widely used for the measurement of many vital signs, such as SpO_2_, heart rate, respiration rate, and BP. The signal measured by this method represents the pulsatile blood volume changes of peripheral microvasculature induced by pressure pulse within each cardiac cycle. Traditionally, the sensing unit is in direct contact with skin. Recent research has shown that sensors can be integrated into daily living accessories or gadgets like ear-ring, glove and hat, to achieve unobtrusive measurement. Various types of PPG measuring devices at different sites of the body are summarized in Table II.

$$\eqalignno{&{\rm PTT} = \left\{{\matrix{{{\displaystyle{{L\sqrt {\rho b} } \over {1 + \exp (bP_i)}}}};\hfill & {h = 0} \hfill\cr {{\displaystyle{{2L} \over {\sqrt {\rho b} gh}}}\ln \left\vert {\displaystyle{{\sqrt {\exp [b(P_i - P_h)] + \exp (- bP_h)} - \sqrt {\exp (- bP_h)} } \over {\sqrt {\exp [b(P_i - P_h)]{\rm + }1} {\rm - }1}}}\right\vert }; \hfill & {{\rm }h{\rm } \ne {\rm }0}\hfill \cr}} \right. &\hbox{(1)}}$$

**TABLE II:** PPG Measuring Devices at Different Sites of the Body

Devices	Location of Sensor /Operation mode	Measured Parameters
PPG ring [27]	Finger /Reflective	Heart rate and its variability, SpO_2_
Pulsear [28]	External ear cartilage /Reflective	Heart rate
Forehead mounted sensor [29]	Forehead /Reflective	SpO_2_
e-AR [30]	Posterior, inferior and anterior auricular /Reflective	Heart rate
IN-MONIT system [31]	Auditory canal /Reflective	Heart activity and heart rate
Glove and hat based PPG sensor [32]	Finger and forehead /Reflective	Heart rate and pulse wave transit time
Ear-worn monitor [33]	Superior Auricular, superior Tragus and Posterior Auricular /Reflective	Heart rate
Headset [34]	Ear lobe /Transmissive	Heart rate
Heartphone [35]	Auditory canal /Reflective	Heart rate
Magnetic earring sensor [36]	Ear-lobe /Reflective	Heart rate
Eyeglasses [37]	Nose bridge /Reflective	Heart rate and pulse transit time
Ear-worn PPG sensor [38]	Ear lobe /Reflective	Heart rate
Smartphone [39]	Finger /Reflective	Heart rate

Recently, Jae et al. [25] proposed an indirect-contact sensor for PPG measurement over clothing. A control circuit was adopted to adaptively adjust the light intensity for various types of clothings. On the other hand, Poh et al. [26] showed that heart rate and respiration rate can be derived from PPG that was remotely captured from a subject's face using a simple digital camera. However, the temporal resolution of the blood volume detected by this method is restricted by the sample rate of the camera (up to 30 frames per second), thus affecting its accuracy.

### C. Model-Based Cuffless BP Measurement

Sphygmomanometer, which has been used over a century for BP measurement, is operated based on an inflatable cuff. Conventional methods such as auscultatory, oscillometric, and volume clamp are not suitable for unobtrusive BP measurement. Pulse wave propagation method is a promising technique for unobtrusive BP measurement. It is based on the relationship between pulse wave velocity (PWV) and arterial pressure according to Moens-Korteweg equation. Pulse transit time (PTT), the reciprocal of PWV can be readily derived from PPG and ECG in an unobtrusive way. Various linear and nonlinear models that expressed BP in terms of PTT have been developed for cuffless BP estimation. The subject-dependent parameters in BP-PTT model should be determined first when using this approach for BP estimation. A very simple way to implement individual calibration is to use the hydrostatic pressure approach, where the subjects are required to elevate their hands to specific heights above/below the heart level [40]. A theoretical relationship between PTT, BP, and height can be written in (1), as shown at the bottom of the page, where $b$ is the subject-dependent parameter characterizing the artery properties, $L$ is the distance traveled by the pulse, $P_{h}$ is the hydrostatic pressure ρ gh, and $P_{i}$ is the internal pressure. Using the proposed model, the individualized parameters in BP-PTT model can be determined from some simple movements. This model-based cuffless BP measurement method can be implemented in a variety of platforms for unobtrusive monitoring, such as the bed cushion and chair, as shown in Fig. 3.

Although the accuracy of this method has been validated in many recent studies [41], there are concerns over the use of PTT as a surrogate measurement of BP. It has been recognized that the major confounding factors in the present relationship of BP and PTT are vasomotor tone and pre-ejection period [42]. New models should be developed to include these confounding factors in order to explore the potential of PTT-based method for unobtrusive BP measurement in future.

### D. Other Unobtrusive Sensing Methods

Strain sensors are commonly used to measure body motion such as respiration, heart sound and BCG. Piezoelectric cable sensor, whose sensing element is piezoelectric polymer, has been used for respiration rate monitoring [43]. Flexible and thin sensors, such as piezoresistive fabric sensors [44] and film-type sensors like polyvinylidenefluoride film (PVDF) and electromechanical film (EMFi) based sensors [45] have been widely used for cardiopulmonary applications due to the easiness of being embedded into clothing or daily objects like chair or bed. Comparative study has been conducted to evaluate the performances of the two different strain sensors in the measurement of body motion [46]. They have shown that only small differences were found in heart rate measured by PVDF-based and EMFi-based sensors especially at supine posture, which was possibly caused by their different sensitivities to different force components.

Inductive/impedance plethysmography is another widely used method for respiratory measurement and has been developed in the forms of clothing and textile belt [47], [48]. Two sinusoid wire coils located at rib cage and the abdomen are driven by a current source that generates high-frequency sinusoidal current. The movement of the chest during respiration causes changes of the inductance of the coils and thus modulates the amplitude of the sinusoidal current, from which the respiratory signal can be demodulated. A recent study compared the performances of four different methods for wearable respiration measurement, including inductive plethysmography, impedance plethysmography, piezoresistive pneumography, and piezoelectric pneumography piezoelectric, and showed that piezoelectric pneumography provided the best robustness to motion artifacts for respiratory rate measurement [43].

Optical fibers have also been adopted for unobtrusive monitoring by embedding them into daily objects or clothes. In contrast to electronic sensors, they are immune to the electromagnetic interference. Recently, fiber Bragg grating has been proposed as a vibration sensor for BCG measurement, based on the fact that the Bragg wavelength is correlated with the grating period in response to the body vibration caused by breathing and cardiac contractions [49]. A pneumatic cushion based on this method has been developed to monitor the physiological conditions of pilots and drivers [49]. D’Angelo developed an optical fiber sensor embedded into a shirt for respiratory motion detection [50].

Other remote sensing methods have also been proposed for unobtrusive physiological measurement, such as frequency modulated continuous wave Doppler radar for BCG measurement [51] and radiometric sensing for body temperature measurement [52].

## III. Wearable Devices

In this section, an overview on the state-of-the-art of wearable systems is presented. Several key enabling technologies for the development of these wearable devices, such as miniaturization, intelligence, networking, digitalization and standardization, security, unobtrusiveness, personalization, energy efficiency, and robustness will be discussed. Sensor embodiments based on textile sensing, flexible and stretchable electronics are also introduced.

### A. Overview of Unobtrusive Wearable Devices

**Fig. 4.**
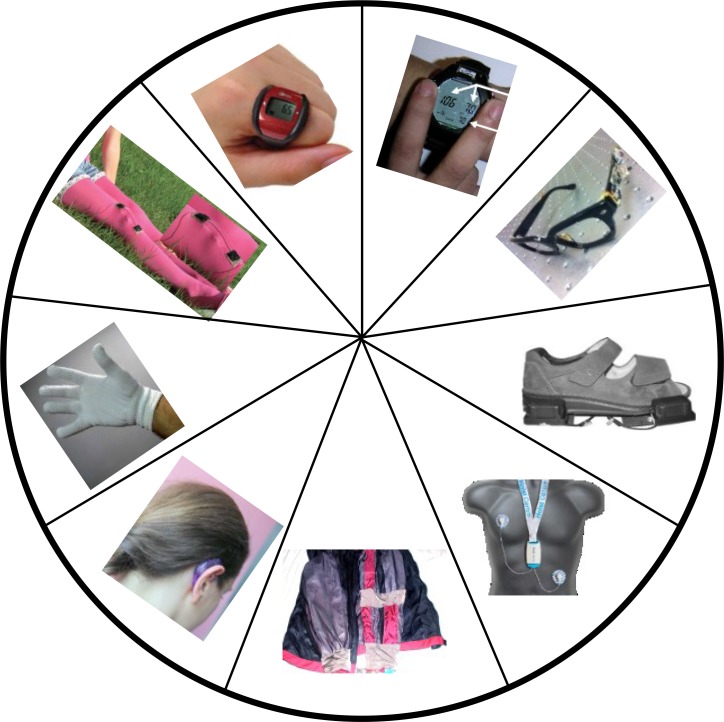
Unobtrusive wearable devices for various physiological measurement developed by different groups: watch-type BP device [3], PPG sensors mounted on eyeglasses [37], motion assessment with sensors mounted on shoes [53], wireless ECG necklace for ambulatory cardiac monitoring (courtesy of IMEC, Netherlands) [54], h-Shirt for BP and cardiac measurements [55], ear-worn activity recognition sensor [60], glove-type pulse oximeter [57], strain sensors mounted on stocking for motion monitoring [58], and ring-type device for pulse rate and SpO_2_ measurement [59].

A variety of unobtrusive wearable devices have been developed by different research teams as shown in Fig. 4: the watch-type BP device [3], clip-free eyeglasses-based device for heart rate and PTT measurement [37], shoe-mounted system for the assessment of foot and ankle dynamics [53], ECG necklace for long-term cardiac activity monitoring [54], h-Shirt for heart rate and BP measurement [55], an ear-worn activity and gait monitoring device [56], glove-based photonic textiles as wearable pulse oximeter [57], a strain sensor assembled on stocking for motion monitoring [58], and a ring-type device for heart rate and temperature measurement [59]. These embodiments are capable of providing measurements ubiquitously and unobtrusively. Many of them already have a variety of applications in healthcare as well as wellness and fitness training. For example, posture and activity monitoring of the elderly and the disabled, long-term monitoring of patients with chronic diseases like epilepsy, cardiopulmonary diseases, and neurological rehabilitation.

Implantable sensors: It is worth noting that sensing devices should not be limited to those designed to be worn on the body. There are increasing numbers and varieties of implantable sensors have been introduced, which are designed to be implanted inside the human body. For example, CardioMEMS recently developed a wireless implantable haemodynamic monitoring system, which can provide long-term measurement of pulmonary arterial pressure of patients with heart failure [61]. A recent clinical study was conducted to validate the safety and effectiveness of this device, and the results showed that the heart-failure-related hospitalization can be significantly reduced in the group with the implant compared to the control group [61]. Another example is the wireless capsule device that can be pinned on the esophageal to measure pH level over a 48-hour period for better diagnosis of gastroesophageal reflux disease [62]. However, the sensor is difficult to be securely attached for the complete sensing periods in some subjects, and premature losing of the capsule has been reported quite often. Other technologies such as the development of mucosal adhesive patches could be a potential solution in this regard. Another important application for mucosal adhesive patches is to design them as drug delivery systems. By placing these devices inside the gastrointestinal tract and with feedback mechanisms installed, the release of drug can be better controlled. This is anticipated to be helpful to the better management of many chronic diseases, such as diabetes and hypertension.

### B. Key Wearable Technologies

In addition to unobtrusive sensing methods mentioned in the last section, some key technologies should also be developed to implement unobtrusive wearable devices for practical use. Zhang's research group summarized these key technologies as “MINDS” (Miniaturization, Intelligence, Networking, Digitalization, Standardization) [63]. The recent development of these technologies will be elaborated as follows together with some other important issues such as unobtrusiveness, security, energy-efficiency, robustness, and personalization.

1) Miniaturization and Unobtrusiveness can enhance the comfortness of using wearable devices, and thus increasing the compliance for long-term and continuous monitoring. Thanks to the rapid development of integrated circuit technologies and microelectromechanical technologies, the size of processing electronics and inertial measurement units (IMUs) has been significantly reduced for wearable applications. For instance, Brigante et al. recently developed a highly compact and lightweight wearable system for motion caption by careful selection of IMUs and optimized layout design [64]. A highly integrated microsystem was designed for cardiac electrical and mechanical activity monitoring, which assembled the multisensor module, signal processing electronics, and powering unit into a single platform with a flexible substrate [65]. In addition to the integrated design and compact packaging, the development of new measuring principle is another way to achieve miniaturization. The aforementioned PTT-based cuffless BP measuring method is a promising substitute for the widely used cuff-based methods in this regard. Further miniaturization can be achieved by using other technologies such as inductive powering where the sensor can be powered partly or completely by RF powering, and the battery can be removed [66].

As mentioned, capacitive coupling sensing is one commonly used method for unobtrusive capturing of bioelectrical signals, like ECG and EEG. Textile sensing is another unobtrusive method for continuously monitoring physiological parameters. A textile-integrated active electrode was presented in [67] for wearable ECG monitoring, which could maintain signal integrity after a five-cycle washing test. In addition, PPG offers a good way for unobtrusive cardiovascular assessment, such as noninvasive measurement of BP based on PTT.

2) Networking and Security: Networking is an integral part of wearable devices to deliver high-efficiency and high-quality healthcare services from the m-health perspective [68]. The term “BSN”—body sensor network—was coined to harness several allied technologies that underpin the development of pervasive sensing for healthcare, wellbeing, sports, and other applications that require “ubiquitous” and “pervasive” monitoring of physical, physiological, and biochemical parameters in any environment and without activity restriction and behavior modification. BSN can be wired (e.g., interconnect with smart fabric) or wireless (making use of common wireless sensor networks and standards, e.g., BAN, WPAN (IEEE 802.15.6), Bluetooth/Bluetooth Low Energy (BLE or Bluetooth Smart), and ZigBee). BSN is presently a very popular research topic and extensive progresses have been made in the past decades. Related to networking, technical challenges include user mobility, network security, multiple sensor fusion, optimization of the network topology, and communication protocols for energy-efficient transmission [69].

In a broader sense, networking for sensing can also include wireless personal area network (WPAN), wireless local area network (WLAN) and cellular networks (3G/4G network, GPRS, GSM) or wireless wide area network (WWAN), which are used to send all the acquired data from wearable devices to data servers for storage and postprocessing. The most critical issue in WPAN/WLAN technologies is to guarantee the quality of service (QoS), which contains latency, transmission power, reliability (i.e., error control) and bandwidth reservation. While for healthcare applications, these issues become more challenging because all the specifications should be satisfied simultaneously. For example, monitoring ECG requires a data rate of tens to hundreds of kilobit/s, end-to-end delay within 2 s and bit error rate of below 10$^{-4}$ [70]. Under some life-critical situations, the compromise between reliability and latency constraint of data transmission is even more prominent. Effective error control schemes have been proposed to address this issue, such as the combined use of feed-forward error control (FEC) and block interleaving in data-link layer [71]. For bandwidth demanding applications, such as two-dimensional (2-D) mode of echocardiograph and ultrasound video transmission, to transmit the data with minimal loss of diagnostic information and in real time, the protocol should be designed to optimize the transmission rate, minimize the latency and maximize the reliability. Various enhanced protocols on rate control have been proposed for these applications, such as a hybrid solution either using retransmission or retransmission combined with FEC techniques depending on the channel conditions [72], adapting the sending rate of source encoder according to the mobile link throughput [73].

**Fig. 5.**
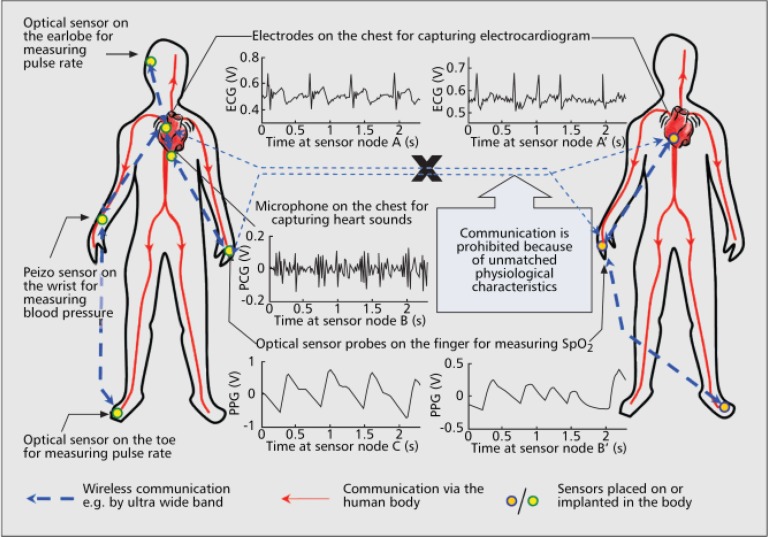
Illustration of using biometrics approach to secure inter-sensors communication for wearable health monitoring applications [77].

Security is a critical issue of networking, especially for medical applications. Without adequate security, the transmitted information is vulnerable to potential threats, such as eavesdropping, tampering, denial of service, which are caused by unauthorized access. The challenging issues to guarantee security lie in three aspects: how to prevent the disclosure of patient's data, who have the right to access the system, and how to protect the privacy of the user [74]. Securing the data transmission between wearable sensors should be the first step. To secure inter-sensor communications, the most important issue is to design a key agreement for encryption. Identity-based symmetric cryptography and asymmetric key cryptography based on elliptic curve have been adopted for BSN [75], [76]. Another novel method is based on biometrics traits. Since the human body contains its own transmission system such as blood circulation system, it is believed that the information from the system itself can be used to secure the information communication between sensors. Meanwhile, since wearable sensors are already there to capture physiological signals, it is convenient to implement encryption schemes using physiological signals in the key agreement. Poon et al. firstly proposed to use the interpulse intervals (IPIs) as the biometric traits to encrypt the symmetric key, as shown in Fig. 5 [77]. The results showed that a minimum half total error rate of 2.58% was achieved when the signals were sampled at 1000 Hz and then coded into 128-bit binary sequences. Subsequently, other similar approaches have been proposed [78]. For example, Venkatasubramanian et al. proposed using frequency domain features of PPG as the basis of key agreement [79]. Furthermore, the complexity of the security scheme should also be considered due to the constraints on the energy budget and computational power of the wearable sensor. Various potential solutions have been developed, such as the data confidential and user authentication schemes with low complexity realized by minimizing the shared keys [80], and mutual authentication and access control scheme based on elliptic curve cryptography with the advantage of energy-efficient [81].

**Fig. 6.**
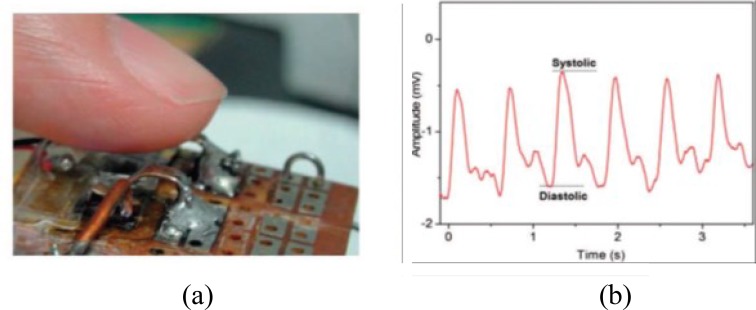
(a) High-sensitivity photo-transistor based on organic bulk heterojunction; (b) PPG signal detected by this photo-transistor [84].

3) Energy-Efficiency and Digitalization: Energy-efficiency is a crucial element of a wearable device that directly affects the design and usability of the device, especially for long-term monitoring applications. There are mainly three strategies to achieve energy-efficient design. The first one is to improve the energy efficiency of the existing energy storage technologies, lithium-ion batteries and supercapacitors. Liu et al. designed a novel structure of ZnCo _2_O_4_-urchins-on-carbon-fibers matrix to fabricate energy storage device that exhibits a reversible lithium storage capacity of 1180 mAh/g even after 100 cycles [82]. Fu et al. first adopted commercial pen ink as an active material for supercapacitors. With simple fabrication, the flexible fiber supercapacitors can achieve areal capacitance of 11.9–19.5 mF cm$^{-2}$ and power density of up to 9.07 mW cm $^{-2}$ [83]. The second is energy-aware design of wearable system. For example, a high-sensitivity near-infrared photo-transistor was recently developed based on an organic bulk heterojunction as shown in Fig. 6(a) [84]. The results showed that the responsivity of this device can achieve 10^5^ A W$^{-1}$. It has been successfully used for PPG measurement as shown in Fig. 6(b) [84]. With the high responsivity, this device allows the use of a low-power light source, and thus reducing the power consumption of the sensor. Power management approaches, such as dynamic voltage scaling, battery energy optimal techniques and system energy management techniques have also been proposed [85]. Last but not the least is to scavenge energy from the environment or human activities, such as the lower-limb motion and vibration, body heat and respiration, which have great potential to become sustainable power sources for wearable sensors. The MIT Media Lab [86] has realized an unobtrusive device that scavenged energy from heel-strike of the user with shoe-mounted piezoelectric transducer. Recently, Leonov [87] has developed a hidden thermoelectric energy harvester of human body heat. It was integrated into clothing as a reliable powering source. For implantable devices, the preferred power solution is wireless powering. Kim et al. have derived the theoretical bound of wireless energy transmit efficiency through tissue. By optimizing the source density, the peak efficiency can achieve 4 × 10$^{-5}$ at around 2.6 GHz, which is 4 times larger than that of the conventional near-field coil-based designs [88].

Digitalization is necessary to enable data analysis and storage after acquiring the analog signals from the body with wearable devices. Due to the rigorous power constraints of wearable devices, the sampling rate should be minimized to save power whilst not compromising the diagnostic accuracy. For example, the sampling rate for ECG should be no less than 125 Hz; otherwise, it will affect the accuracy of measurements [89]. Recently, novel nonuniformly sampling methods have been investigated to compress the representation of ECG signal in which the relevant information is localized in small intervals. One example is the integrate-and-fire sampler that integrates the input signal against an averaging function and the result is compared to a positive or negative threshold as shown in (2),

$$\theta _k = \int_{t_{k + \tau } }^{t_{k + 1} } {x(t)e^{\alpha (t - t_{k + 1})} dt}; \quad \theta _k \in \left\{{\theta _p, \theta _n } \right\}. \eqno{\hbox{(2)}}$$

A pulse will be generated when either of the thresholds is reached. The original signal thus can be represented by a nonuniformly spaced pulse train from the output of the integrate and fire model [90]. This sampler can be implemented by effective hardware to save power and facilitate sensor miniaturization. However, the tradeoff is that exact reconstruction of the original signal is impossible [90]. Nonetheless, some critical information for accurate diagnosis can be preserved. Without reconstruction, a time-based integrate and fire encoding scheme can achieve 93.6% classification rate between normal heartbeats and arrhythmias, which is comparable to other methods [91].

4) Standardization and Personalization: Standardization is a crucial component of any commercial exploitation of wearable devices, as it ensures the quality of the devices and enables interoperability among devices. However, the process of standardization is often very complicated and time consuming, especially in standardizing healthcare devices. Health information can be very different ranging from physiological (ECG, body temperature, oxygen saturation, etc.) to physical (posture, activity, etc.). Manufacturers tend to define their own proprietary formats and communication protocols. Interoperability therefore becomes a major obstacle in integrating devices into healthcare systems. To resolve this issue, standard protocols such as the HL7 reference information model have been proposed for the integration of different sensing modalities. For instance, Loh and Lee proposed the use of an Oracle Healthcare Transaction Base (HTB) system as the platform for integrating different information into a healthcare system [92]. ISO/IEEE 11073 (X73), initially intended for the implementation of Point-of-Care devices, is now extended to Personal Health Devices (PHD) to address the interoperability problem. This standard provides the communication functionality for a variety of wearable devices (i.e., pulse oximeter, heart rate monitor, BP monitor, thermometer, etc.) and standardizes the format of information for plug-and-play interoperability [93]. A recent study has validated the feasibility to apply this standard to wearable and wireless systems in home settings [94]. To meet the needs of some scenarios with limited processing abilities, some amendments have been made based on this standard, such as the adapted data model proposed in [95] and a new method to implement X73PHD in a microcontroller-based platform with low-voltage and low-power constraints [96].

However, very few standards are available for evaluating the accuracy of wearable devices, albeit the necessity of standards to guarantee the reliability of these devices. For example, there are three well-known standards for assessing the accuracy of cuff-based BP measurement devices, i.e., the American Association for the Advancement of Medical Instrumentation (AAMI), the British Hypertension Society (BHS), and the European Society of Hypertension (ESH). On the other hand, Zhang's team has been spear-headed a standard for cuffless BP devices [97]. In this work, a new distribution model of the measuring difference between the test and the reference device was proposed. The cumulative percentages (CP) of absolute differences between the test device and the reference derived by integrating the probability density function over the required range of limits (L) for normal and general t distribution are shown in (3) and (4), respectively:

$$\eqalignno{{\rm CP}_L^g (u,d,L)\! & =\!\! \int\limits_{- L}^L \! {p(x\vert u,d)dx = {1 \over {d\sqrt {2\pi }}}}\! \int\limits_{- L}^L \!{e^{{{- (x - u)^2 \over 2d^2}}} dx} &{\hbox{(3)}}\cr {\rm CP}_L^t (u,d,v,L)\! &= \!\int\limits_{- L}^L \!{p(t\vert u,d,v)dt} \cr &=\! {{d^{- 1} \Gamma ({{v + 1} \over 2}) \over \Gamma ({v \over 2})\sqrt {(v - 2)\pi }}}\!\int\limits_{- L}^L \! {(1 + {{(t - u)^2 \over d^2 (v - 2)}})^{- {{v + 1} \over 2}} dt} \cr &&{\hbox{(4)}}}$$

where $u, d$, and $v$ denote mean, standard deviation, and degree of freedom, respectively. It was found that the agreement between the evaluation results of 40 cuff-based devices by AAMI and BHS protocols was better for the proposed t distribution (82.5%) than normal distribution (50%). The proposed error distribution model was further validated by the goodness-of-fit of 999 datasets obtained from 85 subjects by a PTT-based cuffless BP estimation method to the hypothesized distribution. In addition, this study also proposed new evaluation scales under general t distribution to assess the accuracy of cuffless BP devices, including: mean absolute difference (MAD), root mean square difference (RMSD) and mean absolute percentage difference (MAPD) as shown in (5) and (6) [97]

$$\eqalignno{\hbox{MAD} &= 2s\sqrt {{v \over \pi}} {{\Gamma ((v + 1)/2) \over \Gamma (v/2)(v - 1)}}\left({1 + {{u^2 \over s^2 v}}} \right)^{- {{v - 1} \over 2}} \cr &\quad + \vert u\vert \times \left({1 - I\left({{v \over {v + (u/s)^2}};\;{v \over 2},{1 \over 2}} \right)} \right) &{\hbox{(5)}}\cr \hbox{RMSD} &= u^2 + d^2 &\hbox{(6)}}$$

where $s$ is the scale and equals to $d \times \sqrt {(v - 2)/\pi } (v > 2)$. Based on (3) and (4), a mapping chart to relate the proposed scales with the AAMI, BHS, and ESH evaluation criteria under $t_{4}$ distribution was developed.

On the other hand, personalization of wearable devices is also important. Apart from personalizing the design of the devices, it can be referred to personalizing the sensor calibration, disease detection, medicine, and treatments. Taking BP as an example: individualized BP calibration procedure is required for the PTT based unobtrusive BP measurement to ensure the accuracy. The calibration can be implemented through body movement such as hand elevation [40]. On the other hand, from the perspective of personalized medicine, since BP can be very diverse among individuals, it is necessary to track a person's BP in long-term by wearable devices to establish an individual database for personalized BP management. This can lead to a more precise diagnosis and treatment.

5) Artificial Intelligence and Robustness: Artificial intelligence enables autonomic functions of wearable devices, such as sending out alerts and supporting decision making. Artificial intelligence may also refer to as context-awareness and user-adaption [98]. Various classification methods such as hidden Markov model, artificial neural networks, support vector machines, random forests, and neuro-fuzzy inference system have been extensively applied for various healthcare applications. The need of individualized training data, the lack of large scale studies in real-world situations, and the high rate of false alarms are some of the major obstacles that hinder the widespread adoption of sensing technologies in clinical applications.

One of the major problems for wearable sensors is motion artifacts. The reduction of motion artifact is still a challenging problem for the development of wearable system due to the overlapping of its frequency band with the desired signal. Several methods have been proposed for motion artifacts reduction. For instance, Chung et al. [99] presented a patient-specific adaptive neuro-fuzzy interference system (ANFIS) motion artifacts cancellation for ECG collected by a wearable health shirt. With ANFIS, it was able to remove motion artifacts for ECG in real time. Adaptive filtering is another commonly used method for motion artifacts removal, and algorithms based on it have been applied for various applications. Ko et al. [100] proposed an adaptive filtering method based on half cell potential monitoring to reduce the artifacts on ECG without additional sensors. Poh et al. [36] embedded an accelerometer in an earring PPG sensor to obtain motion reference for adaptive noise cancellation. Although adaptive filtering has shown to be an effective method, it requires extensive processing time, which hinders its real-time applications. Yan and Zhang [101] developed a robust algorithm, called minimum correlation discrete saturation transform, which is more time efficient than discrete saturation transform that used adaptive filter. Other methods, like independent component analysis and least mean square based active noise cancellation, have also been used to remove motion artifacts.

In summary, the main issues to be addressed for the ubiquitous use of wearable technologies can be summarized as “SUPER MINDS” (i.e., Security, Unobtrusiveness, Personalization, Energy efficiency, Robustness, Miniaturization, Intelligence, Network, Digitalization, and Standardization). More attentions should be paid to these aspects for the future development of wearable devices.

### D. Textile Wearable Devices

**Fig. 7.**
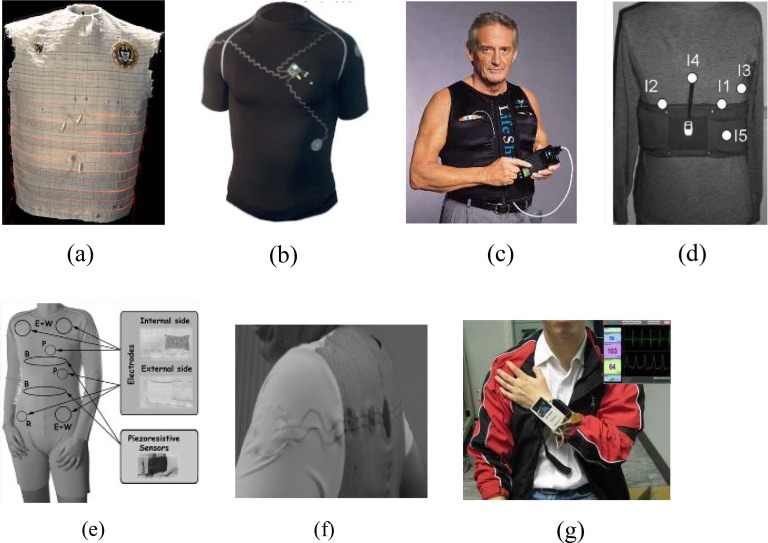
Various wearable garments for physiological and activity monitoring. (a) Georgia Tech Wearable Motherboard (Smart Shirt) for the measurement of ECG, heart rate, body temperature, and respiration rate [105]; (b) the EKG Shirt system that used interconnection technology based on embroidery of conductive yarn for heart rate [106]; (c) the LifeShirt system for the measurement of ECG, heart rate, posture and activity, respiration parameters, BP (peripheral is needed), temperature, SpO _2_ [107]; (d) the ProTEX garment for the measurement of heart rate, breathing rate, body temperature, SpO _2_, position, activity, and posture [108]; (e) the WEALTHY system with knitted integrated sensors for the measurement of ECG, heart rate, respiration, and activity monitoring [44]; (f) the VTAMN system for the measurement of heart rate, breathing rate, body temperature, and activity [109]; (g) the h-Shirt system for the measurement of ECG, PPG, heart rate, and BP [55].

Smart textile technology is a promising approach for wearable health monitoring, since it provides a viable solution to integrate wearable sensors/actuators, energy sources, processing, and communication elements within the garment. Some representative garment-based systems have been developed as shown in Fig. 7.

Despite extensive progresses been made in wearable devices for physiological monitoring, little attention has been paid on the other health information such as biochemical and autonomic information that can provide a more comprehensive picture of the subject's health state. At this point, textile technology provides feasible solutions to the unobtrusive measurement of these new parameters. For instance, a textile-based sweat rate sensor has recently been developed, which measures the water-vapor pressure gradient by two humidity sensors at different distances from skin [102]. Textile-based pH sensor was also proposed using a colormetric approach with pH sensitive dyes and optoelectronics [102]. In addition, Poh et al. developed conductive fabric electrodes for unobtrusive measurement of electrodermal activity (EDA), which can be used for long-term assessment of autonomic nerve activities during daily activities [103]. This textile sensor has been designed into a wrist-worn device that can provide ambulatory monitoring of autonomic parameters for patients with refractory epilepsy. The strong correlation between EDA amplitude and postictal generalized EEG suppression (PGES) duration, an autonomic biomarker of seizure intensity, indicates that EDA can be used as a surrogate measurement of PGES for ambulatory monitoring of epilepsy and thus provides opportunity for the identification of sudden death in epilepsy [104].

### E. Flexible, Stretchable, and Printable Devices

Flexible electronics, where electronic circuits are manufactured or printed onto flexible substrates such as paper, cloth fabrics and directly on human body to provide sensing, powering, and interconnecting functions, have a broad range of biomedical applications. They have enabled the development of wearable devices to be thinner, lighter and flexible. The most common methods to fabricate these flexible sensors and other functional circuits on flexible substrates include: inkjet-printing, screen printing, transfer printing [4], low temperature deposition, etc.

**Fig. 8.**
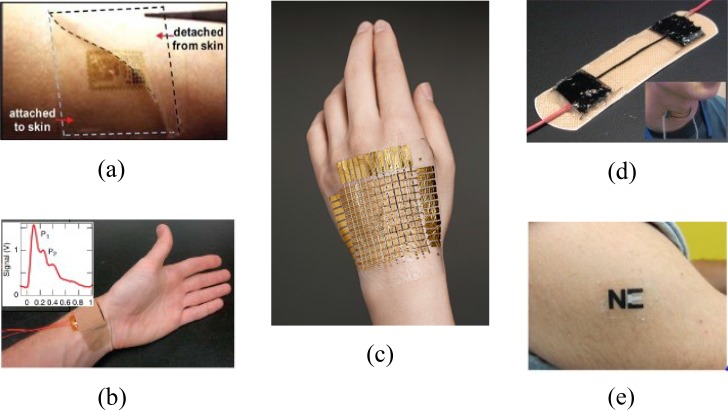
Flexible and stretchable wearable devices for health care applications developed by different groups: (a) multifunctional epidermal electronic device for electrophysiological, temperature, and strain monitoring [4]; (b) a flexible capacitive pressure sensor for radial artery pressure pulse measurement [112]; (c) e-skin with pressure and thermal sensors [113]; (d) a bandage strain sensor for breathing monitoring [58]; (e) electrochemical tattoo biosensors for real-time noninvasive lactate monitoring in human perspiration [116].

Flexible electronics for health monitoring: Fig. 8(b) to (e) shows some cutting-edge flexible wearable systems for health monitoring applications. For example, Bao's team developed a high-sensitivity flexible capacitive pressure sensor with polydimethylsiloxane as the dielectric layer [111]. By microstructuring the dielectric layer and integrating it in a thin-film polymer transistor, it is able to achieve very high sensitivity when driving the flexible pressure-sensitive transistor in the subthreshold regime. This device has been applied to measure a human radial artery pulse wave with high-fidelity as shown in Fig. 8(b) [112]. Other examples include the e-skin with pressure and thermal sensors, and the flexible strain-gauge sensor for breathing monitoring as shown in Fig. 8(c) and (d) [58], [113]. Some recent studies have proposed the fabrication of flexible photo-transistor [114], which can be used to build PPG sensors. These advances will effectively pave the way for unobtrusive physiological measurements in the future. In addition to the applications in physiological and physical monitoring, flexible electronic technology also begins to play a key role in biochemical sensing. A flexible “nano-electronic nose” was developed by Michael et al. by transferring hundreds of prealigned silicon nanowires onto plastic substrate. It would open up new opportunities for wearable or implantable chemical and biological sensing [115]. Another example is the electrochemical tattoo biosensor recently developed by the research team from University of California San Diego, as shown in Fig. 8(e) [116]. It can be easily worn on body for lactate monitoring during aerobic exercise. The performance of this tattoo sensor was evaluated in terms of its selectivity for lactate measurement, the ability of adhering to epidermal surface, and the robustness when exposed to mechanical stretching and bending. The results have showed its potential for unobtrusive and continuous monitoring of lactate during exercise [116].

Flexible sensors with inorganic, organic, and hybrid materials: A variety of organic and inorganic materials have been adopted for the development of flexible sensors. CNT is a very promising carbon based material for the design of flexible devices due to its unique mechanical and electrical properties. A flexible film strain sensor based on CNT has been designed and integrated into fabrics for human motion monitoring [58]. It can endure strain up to 280% and achieve high durability of 10 000 cycles at 150% strain as well as fast response of 14 ms, which might be used for breath monitoring for the early detection of sudden infant death syndrome in sleeping infants. On the other hand, the printable feature and relative ease of fabrication process make organic materials suitable for the implementation of flexible wearable devices. Flexible organic sensors employing thin-film transistor structure for skin temperature measurement and pressure sensing [117], [118] have been proposed.

Due to the complementary benefits of inorganic and organic materials in terms of electrical conductivity, mechanical properties, and low-cost fabrication process, inorganic and organic hybrid materials have great potential for the development of high-performance flexible wearable devices. For example, a high-performance inorganic and organic hybrid photo detector based on P3HT and nanowire was fabricated on a printing paper, which has high flexibility and electrical stability [119]. Lee's team fabricated a flexible organic light emitting diode with modified graphene anode to achieve extremely high efficiency [120]. Xu et al. developed an organic bulk heterojunction based near-infrared photo-transistor [84], which can be modified to a flexible device in the future. This device showed great potential for the design of low power PPG sensor due to its ultrahigh responsivity. Pang et al. developed a flexible and highly sensitive strain-gauge sensor that is based on two interlocked arrays of high-aspect-ratio Pt-coated polymeric nanofibres [121]. This device is featured by its simple fabrication process as well as the ability of measuring different types of mechanical forces with high sensitivity and wide dynamic range. The assembled device has been used to measure the physical force of heart beat by attaching it directly above the artery of the wrist under normal and exercise conditions.

In addition to the aforementioned advances in the design of flexible sensors, flexible technologies also enable other functional circuits such as an inkjet-printed flexible antenna for wireless communication [122], flexible batteries with wireless charger [123], flexible supercapacitors on graphene paper [124] and cloth fabrics [125] for wearable energy storage, etc.

Although significant advances have been made in flexible electronics, only limited applications in unobtrusive health monitoring have been explored until now. Some key challenges still hinder the application of these systems in real life situations for wearable health monitoring, such as engineering new structural constructs from established and new materials to achieve higher flexibility, improving the durability of sensors and interconnects that are frequently exposed to mechanical load, integrating wireless data transmission and powering electronics with the flexible sensors, etc.

Stretchable electronics for health monitoring: Flexible electronics already offers great opportunities in the applications of wearable health monitoring, but is not enough to ensure the conformal integration of the devices with arbitrary curved surfaces. Stretchability, which enables the devices bend to extremely small radius and accommodate much higher strain while maintaining the electronic performance, is a more challenging characteristic and renders wider application possibilities. Some stretchable systems for health monitoring applications have been proposed. One example is the epidermal electronic system developed by Rogers' research group for measuring electrophysiological signals (ECG and EMG), temperature, and strain, as shown in Fig. 8(a). The system consists of multifunctional sensors, processing unit, wireless transmission and powering, which are all mounted on an elastic polymer backing layer. The polymer sheet can be dissolved away, leaving only the circuits adhere to the skin via van der forces [4]. One problem of this device is that it can easily fall off during bathing or exercise. Therefore, they recently developed a new epidermal electronic system that has a total thickness of 0.8 m and can be directly printed onto the skin without the polymer backing. A commercially available spray-on bandage is then adopted as adhesives and encapsulants [110]. Other stretchable systems for biomedical applications have also been reported, such as the CNT-based strain sensor for human motion monitoring [58], electronic skin [113], and other as reviewed in [126].

A number of methods have been proposed for the fabrication of stretchable electronics, such as LED, conductors, electrodes, and thin-film transistors, and can be classified into two groups: exploring innovative structures and engineering new materials. Rogers and coworkers [127] configured the structures into wavy shapes supported by elastomeric substrate. The structure can accommodate up to 20%–30% compressive and tensile strain by changing the wave amplitudes and wavelengths. This buckling approach has also been adopted to fabricate graphene ribbons on stretchable elastomers [128]. Rogers and coworkers also proposed a noncoplanar mesh design with active device islands connected by thin polymer bridges on elastomeric substrates to achieve 140% level of strain [129]. Recently, White et al. [130] demonstrated an ultrathin, flexible and stretchable polymer-based light emitting diode (PLED), which can be fabricated with very simple processing method. By pressed onto a prestrained elastomer, the ultrathin PLED formed a random network of folds when releasing the strain on the elastomer. The experiment showed that the ultrathin PLED can be stretched up to 100% tensile strain. Meng et al. [131] designed and fabricated the assembled fiber supercapacitors composed of two intertwined graphene electrodes that showed highly compressible (strain of 50%) and stretchable (strain of 200%) properties, and can be easily woven into textile for wearable applications. New materials can also provide stretchability for electronics. Someya's group fabricated a composite film by uniformly dispersing single-walled CNTs in polymer matrices [132]. Other stretchable electronics based on nanocomposites such as gold nanoparticle-polyurethane composite as conductor [133], graphene oxide nanosheet-conducting polymer as electrode [134] have also been proposed recently. These nanocomposites achieved both good electronic performance and mechanical robustness. These recent advancements in stretchable electronics will facilitate the adoption of these technologies in healthcare applications.

To summarize, with the advances in material, textile, power scavenging, sensing and wireless communications, there has been a rapid increase in the number of new wearable sensing devices launched in the consumer market for sports, wellbeing and healthcare applications. Considering the growing interests in these research areas, the number and variety of sensors will continue to grow in a very rapid rate. In addition, following the current trend of sensing development, new type of implantable sensors, such as transient and zero-power implants, could soon become reality. Standards have been emerged aiming to standardize these devices as well as the wireless communication and the exchange of information among devices and systems.

## IV. Sensing Informatics: Data Fusion and Big Data

The development of sensing technology has largely increased the capability of sensor to acquire data, and multiple sensors are expected to provide different viewpoints of the health status of the patients. However, multisensor data fusion is one great challenge because heterogeneous data need to be processed in order to generate unified and meaningful conclusion for clinical diagnosis and treatment [135]. The fusion of sensing data with other health data such as imaging, bio-markers and gene sequencing and so on is even more challenging. The definitions of data fusion are different in the literature. In [136], data fusion is defined as a “multilevel, multifaceted process handling the automatic detection, association, correlation, estimation, and combination of data and information from several sources”. A comprehensive review and discussion of data fusion definitions are presented in [137]. We propose the definition of data fusion as: “to develop efficient methods for automatically or semi-automatically translating the information from multiple sources into a structured representation so that human or automated decision can be made accurately”. Data fusion is definitely a multidisciplinary research area, which has integrated many techniques, such as signal processing, information theory, statistical estimation and inference, and artificial intelligence. In this section, we will discuss multisensor fusion methods and the fusion of sensing data with other types of health data for clinical decision support. At the end of this section, we will provide a critical on the impact of big data in the healthcare area.

### A. Data Fusion

Since most of health data are accompanied with a large number of noisy, irrelevant and redundant information, which may give spurious signals in clinical decision support, it is therefore necessary to filter the data before fusion. To address this issue, ranked lists of events or attributes clearly relevant to clinical decision-making should be created [138]. Temporal reasoning method has been suggested for detecting associations between clinical entities [139]. More sophisticated methods such as contextual filters [140], statistical shrinkage towards the null hypothesis of no association [141] were also proposed. How to filter information is definitely clinically meaningful, and it would become more and more important but challenging due to the ever-increasing data types and volumes.

Multisensor fusion: A number of algorithms have been proposed in the literature for multisensor data fusion. Due to heterogeneous nature of the data that need to be combined, different data fusion algorithms have been designed for different applications. These algorithms can utilize different techniques from a wide range of areas, including artificial intelligence, pattern recognition, statistical estimation, and other areas. Health informatics has naturally benefited from these abundant literature. For instance, these fusion algorithms can be categorized into the following groups:

1) Statistical approach: weighted combination, multivariate statistical analysis and its most state-of-the-art data mining algorithm [142]. Among all the statistical algorithms, the arithmetic mean approach is considered as the simplest implementation for data fusion. However, the statistical approach might not be suitable when the data is not exchangeable or when estimators/classifiers have dissimilar performances [143].
2) Probabilistic approach: Maximum likelihood methods and Kalman filter [144], probability theory [145], evidential reasoning and more specifically evidence theory are widely used for multisensor data fusion. Kalman filter is considered as the most popular probabilistic fusion algorithm because of its simplicity, ease of implementation, and optimality in a mean-squared error sense. However, Kalman filter is inappropriate for applications whose error characteristics are not readily parameterized.
3) Artificial intelligence: artificial cognition including genetic algorithms and neural networks. In many applications, the later approach serves both as a tool to derive classifiers or estimators and as a fusion framework of classifiers/estimators [142], [143].

Fusion of sensing data with other health data: It is of vital importance to integrate sensing data with other health data, including genetic, medication, laboratory test, imaging data and narrative reports that provide context information for clinical diagnosis. Electronic health record (EHR) reposits these health data in a computer-readable form, which can be used for clinical decision support [146]. The heterogeneous contents and huge size of EHR bring great challenges for the fusion of these data. Bayesian network, neural networks, association rules, pattern recognition and logistic regression have been used to extract knowledge from EHR for patient stratification and predictive purposes in the past years. These methods offer significant advantages to traditional statistical methods because they are able to identify more complex relationships among variables. A comparison has been made among the three different classification methods in terms of their performance in predicting coronary artery disease [147]. It was found that the multilayer perceptron based neural network method showed the best performance in prediction [147].

Although these studies have shown promising results about the effectiveness of these computerized methods to be a complementary tool of the present statistical models for medical decision support, these methods still suffer from some problems, such as over fitting, computationally expensive, and some of them are difficult to be interpreted by experts. Unlike these completely data-driven approaches, fuzzy cognitive map (FCM) is a logical-rule-based method constructed from human experts’ knowledge. It models the decision system as a collection of concepts and causal relationships among these concepts using fuzzy logic and neural networks [148]. It, therefore, can be interpreted by experts and provides transparent information to the experts. However, it could be subjective and may not be reliable by solely relying on experts’ knowledge to generate the rules. A new FCM framework was proposed recently [149], which extracts fuzzy rules from both experts’ knowledge and data using rule extraction methods. This novel framework for FCM system has been used in the radiation therapy for prostate cancer.

**Fig. 9.**
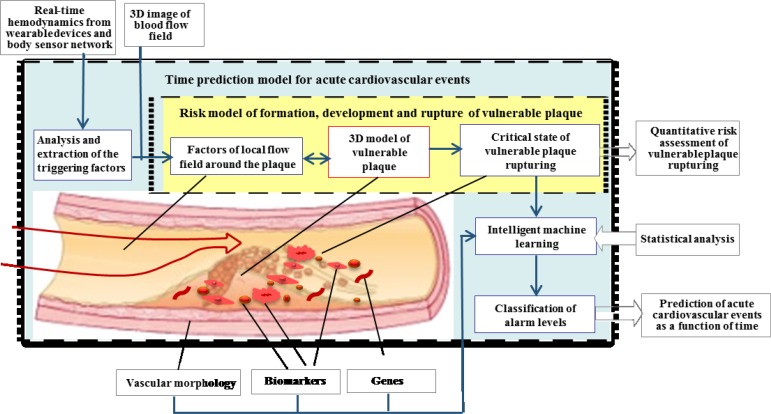
The framework for the quantitative assessment of the risk of acute cardiovascular events. Reproduced from [151].

The early prediction of cardiovascular diseases (CVDs) has been a very popular yet challenging research topic, and many fusion works have been conducted in this area. For instance, in an European Commission funded project, euHeart Project, a probabilistic fusion framework was developed to assimilate different health data across scales (e.g., protein level ion channels flux and whole organ deformation) and functions (e.g., mechanical contraction and electrical activation), into a personalized multiphysics cardiac model by minimizing on the discrepancy between the measurements and the estimating derived from the computational model and then to discover new knowledge using the personalized model [150]. By integrating continuous and real-time sensing data from unobtrusive/wearable devices with the existing health data, the real-time prediction of acute CVD events may become possible. Zhang's team has proposed a personalized framework for quantitative assessment of the risk of acute CVD events based on vulnerable plaque rupturing mechanism as shown in Fig. 9 [151]. This framework does not only take traditional risk factors, sensitive biomarkers, blood biochemistry, vascular morphology, plaque information, and functional image information as inputs of the prediction model, but also gathers physiological information continuously from unobtrusive devices and body sensor networks as the trigger factors targeting for real-time risk assessment of acute cardiovascular events.

### B. Impact of Big Health Data

With the increasing number of sensing modalities and low cost sensing devices becoming more accessible to wider populations, the amount and variety of health data have increased rapidly, health data are therefore considered as big data. For example, if we collect the health data related to cardiovascular system, including ECG, PPG, BP waveform, BCG, EEG, EMG, PTT, cardiac output, SpO_2_, body temperature, and imaging information from computed tomography, magnetic resonance imaging, ultrasound imaging and so on, the size of data collected from all the people in the Southern China, from all the people in the China, and from all over the world, are over 4 zettabytes, 40 zettabyes, and over 200 zettabytes per year, respectively. As of year 2012, the size of data sets that are feasible to process in a reasonable amount of time were in the order of exabytes, which means the size of health data is over one thousand times larger than the current limits. Based on the “4 V” definition of big data by Gartner, i.e., volume, velocity, variety and value, the characteristics of health data can be summarized as “6 V”: vast, volume, velocity, variety, value, variation. Vast is the core feature of health data, i.e., the volume, velocity, variety, value and variation are vast. Volume refers to the size of the information. Because health data are collected from over a huge amount of people simultaneously, the velocity of the collection of health data can be extremely high. A variety of health data from genetic or molecular level like gene sequences and biomarkers to system levels such as physiological parameters and medical imaging data are now available. If we can build up a systematic analysis framework, we can definitely mine knowledge of great value from the health data. Since the biological system of the human being is dynamic and evolving, the health data must be variational.

Due to the ongoing developments of network, mobile computing and computer storage, the storage and retrieval of big health data have attracted great attentions. With the increasing use of various kinds of long-term monitoring devices, there is an urgent demand for the intelligent management of big health data. A large number of cloud-based storage and retrieval systems are emerging in recent years. Through outsourcing the big health data, storage-as-a-service is an emerging solution to alleviate the burden and high cost of large local data storage. Some new storage strategies have been proposed, such as storage consolidation and virtualization to optimize the tradeoff between computation and storage capacity [152]. Retrieving specific information from the cloud-based health data is also very challenging. Query accuracy and time efficiency are two important metrics to evaluate the performance of the retrieval system. Extensive efforts have been made on optimizing the query complexity and expansion strategies to improve the performance of the retrieval systems [153], [154]. Recently, a standards-based model has been proposed to provide a feasible solution for the management (i.e., collection, storage, retrieval and sharing) of patient-generated personal health data in EHR [155]. This model described the required components and minimal requirements for data collection and storage, as well as the method for the exchange of health data between patients and EHR providers.

Up to date, a number of healthcare applications based on cloud computing technology have been reported, such as storage and management of EHRs [156], automatic health data collection in health care institutions [157], intelligent emergency health care [158], radiotherapy, etc. It will further provide opportunities in clinical decision support (particularly for chronic diseases), public health management (e.g., controlling of infectious diseases), development of new drugs, etc [159], [160].

Although many cloud-based storage and retrieval systems have recently been proposed, fusion techniques for analyzing big health data are still in the conceptual stage, we believe that with the advances of computing power as well as the increasing availability of more and more interoperable electronic health records in the healthcare ecosystems, the intelligent utilization of big health data will eventually become feasible in the near future.

## V. Conclusion and Future Development

In this paper, an overview of unobtrusive sensing platforms either in wearable form or integrated into environments is presented. Although significant progresses in developing these systems for healthcare applications have been made in the past decades, most of them are still in their prototype stages. Issues such as user acceptance, reduction of motion artifact, low power design, on-node processing, and distributed interference in wireless networks still need to be addressed to enhance the usability and functions of these devices for practical use. Due to the multidisciplinary nature of this research topic, future development will greatly rely on the advances in a number of different areas such as materials, sensing, energy harvesting, electronics and information technologies for data transmission and analysis. In the following, we put forward some promising directions on the development of unobtrusive and wearable devices for future research:

1) To develop flexible, stretchable and printable devices for unobtrusive physiological and biochemical monitoring: Research on a variety of semiconductor materials, including small-molecule organics and polymers, inorganic semiconducting materials of different nanostructures, like nanotube, nanowire, and nanoribbons and hybrid composite materials, could prosper the design of flexible and stretchable sensors with high optical, mechanical and electrical performance. With the development of flexible, stretchable and printable electronics, wearable devices would evolve to be multifunctional electronic skins or skin-attachable devices, which are very comfortable to wear. Meanwhile, other applications of flexible and stretchable devices in wearable health monitoring should be explored.
2) To develop wearable physiological imaging platforms, especially unobtrusive ones: Physiological information provided by the present wearable systems are generally in one-dimensional (1-D) format. The extension from 1-D to 2-D by including spatial information would be desirable to provide more local information. Compared to the existing noninvasive imaging modalities (e.g., magnetic resonance imaging, computed tomography, ultrasound, etc.), wearable physiological imaging devices would be much faster to achieve high temporal resolution images, which is expected to provide additional clinical and health information for early diagnosis and treatment of diseases.
3) To develop wearable devices for disease intervention: The existing wearable devices are mainly designed for the continuous monitoring of a person's physiological or physical status. For future development, wearable devices should also play a role in disease intervention through integration with actuators that are implanted inside/on the body. One well-known example is the wearable artificial endocrine pancreas for diabetes management, which is a close-loop system formed by a wearable glucose monitor and an implanted insulin pump. Wearable drug delivery systems for BP management can also be developed in the future.
4) To develop systematic data fusion framework: To integrate the multimodal and multiscale big health data from sensing, blood testing, bio-marker detection, structural and functional imaging for the quantitative risk assessment and the early prediction of chronic diseases.

It is believed that with these future developments, unobtrusive and wearable devices could advance health informatics and lead to fundamental changes of how healthcare is provided and reform the underfunded and overstretched healthcare systems.

## Supplementary Material

Color versions of one or more of the figures in this paper are available online at http://ieeexplore.ieee.org.
